# Topics Analysis of Reddit and Twitter Posts Discussing Inflammatory Bowel Disease and Distress From 2017 to 2019

**DOI:** 10.1093/crocol/otab044

**Published:** 2021-07-07

**Authors:** Jacob A Rohde, Adams L Sibley, Seth M Noar

**Affiliations:** 1 Hussman School of Journalism and Media, University of North Carolina, Chapel Hill, North Carolina, USA; 2 Department of Health Behavior, Gillings School of Global Public Health, University of North Carolina, Chapel Hill, North Carolina, USA; 3 Lineberger Comprehensive Cancer Center, University of North Carolina, Chapel Hill, North Carolina, USA

**Keywords:** inflammatory bowel disease, social media, Twitter, Reddit, distress

## Abstract

**Background:**

Social media platforms are popular tools for people with inflammatory bowel disease (IBD) to seek support. In the current study, we sought to examine and characterize IBD and distress discourse on public social media platforms. Our goal was to identify topics associated with these online discussions.

**Methods:**

We collected public social media posts about IBD and distress from Reddit (*N* = 40 625) and Twitter (*N* = 40 306) published between September 2017 and August 2019. We created a term-based dictionary to characterize posts based on 8 different, nonmutually exclusive topics: (1) symptoms, (2) medication, (3) nutrition, (4) procedures, (5) marijuana, (6) stigma, (7) ostomy, and (8) intimacy. We used descriptive statistics to characterize the frequency and order the prevalence of the topics on the 2 platforms, and to assess topic co-occurrences among posts.

**Results:**

Most Reddit (79%) and Twitter (56%) posts mentioned at least 1 IBD topic. The order of topic prevalence was the same for the 2 platforms. Symptoms was the most mentioned topic (Reddit: 57%, Twitter 36%), followed by medication (Reddit: 30%, Twitter 11%), and nutrition (Reddit: 27%, Twitter 9%). Intimacy was the least mentioned topic (Reddit: 2%, Twitter: <1%). Topic co-occurrences varied by platform. Most Reddit posts (57%) mentioned at least 2 IBD topics, whereas only 27% of tweets mentioned multiple IBD topics.

**Conclusions:**

This study contributes to a growing literature examining how IBD is discussed on social media—specifically, in distress-related contexts on Reddit and Twitter. These cross-platform findings highlight important areas potentially associated with IBD-related distress, which could help facilitate future support.

## Introduction

Inflammatory bowel disease (IBD) is a highly stigmatized gastrointestinal (GI) disease.^1–3^ Evidence from a general population survey suggests people attribute more stigma to people with IBD than those with diabetes or HIV/AIDS.^4^ Such stigma can have detrimental effects on psychological well-being and may cause some people with IBD to avoid going out in public and socializing with others.^3,5,6^

As a result of such stigma, many people with IBD use online social media platforms, such as Reddit and Twitter, to cope with the various aspects of their illness.^7^ Indeed, IBD is frequently discussed on social media.^8,9^ Evidence shows that many individuals with IBD use social media to seek informational support from others about their disease.^10–13^ Examples of such support include learning about the efficacy of IBD treatments, best practices for maintaining a nutritional diet, and information about the many different GI-related surgeries and procedures that some people with IBD undergo (eg, colonoscopies).

People with IBD also tend to use social media for emotional support purposes, such as uplifting others with IBD who feel down or validating one another’s disease-related struggles.^7,10,12–14^ Importantly, online emotional support can provide people with IBD—particularly those experiencing disease-related distress—with critical education and emotional guidance about how to better cope with disease symptoms.

Finally, some research suggests people with IBD use social media for their technological affordances.^15^ A recent qualitative study concluded that social media offer people with IBD different therapeutic features such as the means to connect with others with IBD, or the ability to share disease-related stories and narratives.^15^ These findings are corroborated by results from other studies indicating that people with IBD often use social media to network with likeminded individuals,^16^ or to reconstruct or develop new social identities as a means of self-expression.^7^

Although a great deal of literature has examined IBD patients’ use of social media for seeking support, the behavioral and psychological effects of this type of purposive social media use is a relatively new research area. One recent study found that not only did health information about IBD on social media improve Crohn’s disease patients’ perceived levels of social and peer support, but that such support had a further mediating impact on a number of important illness outcomes, such as symptom management and treatment understanding.^17^ In other words, the support afforded by social media could serve to improve disease self-management outcomes for people with IBD.

While the literature shows that individuals with IBD use social media for a variety of supportive purposes, and that such support can improve certain patient outcomes, no systematic work that we are aware of has sought to leverage existing IBD-related social media discussions to identify *specific* areas in which those who are struggling with IBD may need support. In other words, are some aspects of living with IBD more stressful than others, such as treatment adherence or symptom management? It is important to identify these potential areas in order to better understand this population’s priority concerns. Moreover, such information would be useful for future health intervention efforts aimed at providing support to people with IBD.

For the current study, we aimed to identify key topics associated with IBD through analysis of public discourse on social media. More specifically, we sought to examine the prevalence of different IBD topics among posts discussing IBD and distress (eg, depression, anxiety, symptom struggles) in order to better understand what factors may be associated with or contribute to experiences of distress in this population.

## Materials and Methods

### Data Collection

We collected data from the social media platforms Reddit and Twitter. Reddit is a forum-based, pseudo-anonymous social media platform founded in 2005.^18^ Individuals on Reddit interact with one another via user-created, topic-based communities called “subreddits.” Subreddits are often monitored by 1 or more moderators to ensure that users posting in the subreddit are discussing community-relevant content. Reddit allows users to post long-form submissions up to 40 000 characters.

Twitter, on the other hand, is a microblogging social media platform founded in 2006. User submissions to Twitter are referred to as “tweets.” One important feature of Twitter is the hashtag, which is a convention used to categorize and track trending topics across the platform. Hashtags are denoted by the “#” symbol before a word or phrase in a tweet. For example, “#crohns” represents the indexing of a conversation on the topic of Crohn’s disease. In contrast with Reddit, Twitter only allows users to post content up to 280 characters long (though users can link multiple tweets and responses together in a single thread).

We chose these platforms because evidence shows people with chronic disease—including those with IBD—use them for discussing health-related topics, and for spreading public awareness about IBD.^8,19–21^ As of 2019, approximately 22% and 11% of American adults have ever used Twitter and Reddit, respectively, suggesting they are modestly popular online platforms.^22^

For the Reddit data, we downloaded all available Reddit submissions and comments about IBD from PushShift—a publicly available archive of Reddit posts dating back to 2005.^23^ Reddit *submissions* constitute original posts published on a subreddit whereas Reddit *comments* constitute responses to published submissions. The data downloaded from PushShift also included several meta-data variables, such as publication timestamps (ie, the date and time that a social media post was published) and what subreddit each post was published in. For the Twitter data collection, we used the Twitter Intelligence Tool (TWINT)^24^ to download all publicly available tweets about IBD. Similar to the Reddit data, we also collected relevant tweet meta-data, such as usernames and timestamps, for descriptive purposes.

For both platforms, we extracted study-relevant social media posts based on whether they contained at least one of the following IBD keywords: “crohn,” “ibd,” “colitis,” “inflammatory bowel disease,” “ileitis,” and “ileoceceal.” These keywords represent individual categories or umbrella terminology associated with IBD. It should also be noted that collection criteria were case insensitive. That is, “ibd” and “IBD” equally met eligibility criteria. In all, the collection period of both Reddit and Twitter data sets spanned from September 2017 to August 2019. This collection provided a robust indication of how IBD was discussed on these 2 platforms over a long period of time, thus reducing potential cross-sectional analytic biases, such as large spikes of discussions about IBD in a short span which can occur during coordinated IBD awareness events (eg, World IBD day).

We applied several data cleaning procedures to the 2 data sets. For example, we removed “retweets,” which are a feature on Twitter that allows users to repost tweets published by other users on their own profile, to ensure we only analyzed original content. We also removed duplicate posts that were published by the same user. Finally, we removed posts that were irrelevant to IBD or to the purposes of the current study. For example, we eliminated several posts discussing *Investor’s Business Daily* (which is often shortened to “IBD”), as well as posts discussing IBD in the context of animal diagnoses (eg, “I just learned my dog has IBD”). After applying all data cleaning procedures, the sizes of the Reddit and Twitter data sets were *N* = 100 417 and *N* = 260 110, respectively. It should be acknowledged that the social media data under investigation in this study represent general discussions of IBD on these 2 platforms, meaning the data were not limited to posts by users diagnosed with IBD.

### Extracting Distress-Related Social Media Posts

We extracted only the Reddit and Twitter posts containing distress-related content to include in the analyses for the current study. For this process, we developed a list of approximately 300 keywords associated with distress, such as “afraid” and “scared,” and then filtered for posts containing at least one of those words. Relevant phrases or word co-occurrences (ie, bigrams) were also considered in the filtering process. Example word co-occurrences were “feel alone” and “hard to manage.” Throughout this process, we tested the relevancy of the results of the distress filter by iteratively extracting and reviewing samples of posts that were identified by the computer. This step was important as it revealed additional keywords that needed to be added to the filter, as well as keywords that needed to be modified or removed altogether.

Next, we extracted a random sample of 200 posts (a mix of both data sets), and 2 human coders independently characterized posts as pertaining to distress or not. The purpose of this step was to ensure that the posts being identified by the computer were actually relevant to IBD and distress. Intercoder reliability between the 2 human coders was acceptable (*α* = .90; 95% agreement). We also compared the human coding results (after normalizing discrepancies between the 2 independent coders) against the results of the computer classification. Results from this reliability check (ie, human vs computer) were also acceptable (*α* = .90; 95% agreement). No additional changes were made to the distress filter after achieving acceptable reliability.

The final iteration of the filter extracted *N* = 40 625 Reddit posts (including both submissions and comments) and *N* = 40 306 tweets. These 2 data sets were used in all subsequent analyses in the current study.

### Identifying Dictionary Topics

We created a term-based computational dictionary using Python (version 3.8.0) to categorize the Reddit and Twitter posts based on the following 8 IBD topics: (1) symptoms, (2) medication, (3) nutrition, (4) IBD procedures, (5) marijuana, (6) stigma, (7) ostomy, and (8) intimacy. We chose these topics because literature suggests they are important concerns for people with IBD.^25–28^ In addition, some of the IBD topics were chosen based on preliminary descriptive and inferential analyses (eg, word frequencies) of the data. For example, “FODMAP” and “weed” were common words in both data sets, suggesting that “nutrition” and “marijuana” were likely both important topics related to IBD and distress discourse on social media. Going forward, we will italicize all in-text mentions of the IBD topics for clarity.

Notably, the 8 IBD topics are *not* mutually exclusive. That is, a single social media post from either Reddit or Twitter could have been categorized as discussing multiple IBD topics, such as *medication*, *symptoms*, and *nutrition* (eg, “My medication is not helping my Crohn’s disease symptoms so I may need to start cutting back on fiber in my diet”). This categorization approach gave us the ability to examine whether the IBD topics co-occurred with one another and, if so, to what extent.

### Validating Dictionary Topics

Similar to the method used for assessing the relevancy of distress-related posts, we iteratively tested the topics dictionary on the Reddit and Twitter data sets and reviewed samples of the results—modifying, adding, and removing dictionary keywords as needed for each of the 8 IBD topics. This process revealed some posts that were inaccurately labeled by the dictionary. For example, posts about how people were tired of explaining their disease to others were classified under *symptoms* because of the word “tired.” To control for inaccurate classifications, we created a second term-based dictionary that categorized posts containing false positive keywords. We then eliminated the false positive results from the results of the main dictionary analysis. See Supplementary Material for the full list of keywords of each IBD dictionary topic, as well as corresponding false positive terms.

Once the keywords in both the main IBD topics and false positive dictionaries were sound, we extracted a purposive sample of 960 social media posts (a mix of both Reddit and Twitter posts) to compute intercoder reliability and to evaluate the results of the computer classification. We chose this number of posts to ensure there was appropriate representation among each of the IBD topics in the sample. More specifically, we ran the dictionary analysis for both the Reddit and Twitter data sets. Using the computer classification results, we extracted 120 random posts for each IBD topic—60 of which were classified by the computer as containing a respective topic and 60 that were classified as not containing the topic. It is important to note that while the minimum number of posts classified as one of the topics in the reliability analysis data set would be 60, that does not mean this is the maximum number of posts (due to topic co-occurrences). For example, extracting posts that were not classified as containing discussions about IBD and *intimacy* still could have contained mentions of other topics, such as *symptoms* or *medication*.

These 960 posts were aggregated together and then assessed independently by 2 human coders, with each coder classifying the individual posts based on the 8 IBD topics. The final dictionary exhibited acceptable reliability across all IBD topics between the 2 human coders (mean topic reliability: *α* = .92; range: *α* = .85–.96), as well as between the human coders (after normalizing coder discrepancies) and the computer dictionary analysis results (mean topic reliability: *α* = .85; range: *α* = .77–.95).

### Data Analysis

We used descriptive statistics to characterize the social media data, and to rank order popular subreddits (Reddit data set only) and hashtags (Twitter data set only). To contextualize the IBD topics, we extracted example Reddit and Twitter posts categorized under each topic and paraphrased the content of the posts. We chose not to publish verbatim posts to protect the privacy and confidentiality of the users.^29^ All descriptive analyses were computed using Python (version 3.8.0).

We applied network statistics to assess IBD topic co-occurrences (eg, 2 or more IBD topics present in a single post) and topic degree centrality for the Reddit and Twitter data sets. We created network sociograms to visualize the extent of topic co-occurrences using the igraph package in R (version 3.6.3).^30^ The nodes in the sociograms represent each of the 8 IBD topics. The sizes of the nodes are weighted based on their in-degree centrality score across the full topic co-occurrences network, with larger nodes indicating a higher in-degree centrality score. Similarly, the connections (ie, edges) between IBD topics in the sociograms are weighted based on the frequency of co-occurrences between topics, with larger lines indicating more co-occurrences.

### Ethical Considerations

All methods and procedures used in this study were approved by the University of North Carolina Institutional Review Board.

## Results

### Characteristics of the Social Media Data

General characteristics of the Reddit and Twitter data are in Table 1. There were 18 893 and 20 665 unique users who posted content in the Reddit and Twitter data sets, respectively, with users across both platforms posting an average of 2 times. Roughly 4% of Reddit posts and 25% of tweets contained at least 1 hyperlink (ie, web URL).

**Table 1.** Characteristics of the Reddit (*N* = 40 625) and Twitter (*N* = 40 306) data sets

	Reddit	Twitter
	*n* (%) or *M* ± *SD*	*n* (%) or *M* ± *SD*
Users		
Unique users^a^	18 891	20 665
Average posts per user	2.12 ± 5.03	1.95 ± 4.71
Hyperlinks		
Posts containing hyperlinks	1793 (4%)	10 177 (25%)
Total number of hyperlinks	3698	14 325
Type of Reddit post		
Comment	38 633 (95%)	—
Submission	1992 (5%)	—
Subreddits (Reddit)		
Unique subreddits	2534	—
Posts in IBD subreddits	13 303 (33%)	—
Posts in non-IBD subreddits	27 322 (67%)	—
Hashtags (Twitter)		
Total number of hashtags		51 065
Unique hashtags	—	9043
Tweets with 1 or more hashtag	—	12 363 (31%)
Average hashtags per post	—	1.27 ± 3.03

Abbreviations: IBD, inflammatory bowel disease; *M*, mean; *N*, total sample; *n*, sample size; *SD*, standard deviation.

^a^Only includes users who posted at least once in the data, not those who were mentioned by others in posts; average length of tweets in words: *M* = 33, *SD* = 13; average length of Reddit comments in words: *M* = 143, *SD* = 150; average length of Reddit submissions in words: *M* = 208, *SD* = 210.

For Reddit, 95% of posts were comments and 5% were original submissions. These posts were published in 2534 different subreddits. The most popular subreddits based on frequency were r/CrohnsDisease (26%; Table 2), r/AskReddit (13%), and r/UlcerativeColitis (3%). For Twitter, there were 9120 unique hashtags in the data set and 31% of tweets contained at least 1 hashtag. Overall, the most used hashtags were #crohns (11%), #ibd (8%), and #colitis (3%); though, popular non-IBD hashtags included #anxiety and #pain (both 1%).

**Table 2.** Top 10 subreddits and hashtags in the Reddit (*N* = 40 625) and Twitter (*N* = 40 306) data sets

	*n* (%)
Subreddit (Reddit)	
CrohnsDisease	10 529 (26%)
AskReddit	5098 (13%)
UlcerativeColitis	1172 (3%)
IBD	1030 (3%)
ibs	751 (2%)
AskDocs	532 (1%)
Trees	399 (1%)
AmItheAsshole	296 (1%)
relationships	287 (1%)
ostomy	282 (1%)
Hashtag (Twitter)	
#crohns	5490 (11%)
#ibd	4085 (8%)
#colitis	1766 (3%)
#crohnsdisease	1546 (2%)
#ulcerativecolitis	1184 (2%)
#chronicillness	1009 (1%)
#anxiety	591 (1%)
#pain	526 (1%)
#spoonie	497 (1%)
#depression	497 (1%)

Abbreviations: IBD, inflammatory bowel disease; *N*, total sample; *n*, sample size; hashtag text was normalized (eg, lowercasing text, removing special characters) prior to analysis.

### Prevalence of IBD Topics on Reddit and Twitter

Paraphrased social media posts contextualizing each of the 8 IBD topics are in Table 3. The frequencies and proportions of the IBD topics are in Table 4. Most Reddit (79%) and Twitter (56%) posts contained at least 1 IBD topic. The order of topic prevalence was the same for the 2 platforms. That is, *symptoms* was the most prevalent topic (Reddit: 57%, Twitter: 36%), followed by *medication* (Reddit: 30%, Twitter: 11%), *nutrition* (Reddit: 27%, Twitter: 9%), and *IBD procedures* (Reddit: 17%, Twitter: 6%). All other topics were below 10% for both data sets, with *intimacy* ranking as the least prevalent IBD topic (Reddit: 2%, Twitter: <1%).

**Table 3.** Example social media posts by IBD topic

IBD topic	Example social media posts by topic
Symptoms	• New to Crohn’s & need advice on flare ups!
	• IBD sucks so much. My stomach has been in pain all day today.
Medication	• Anyone with Crohn’s using Stelara? Does it cause psoriasis? I’m getting scared, because I saw red stuff on my face.
	• My Prednisone steroids are done. Unfortunately, so have the effects of using them. Talking to my IBD nurse soon.
Nutrition	• I needed to stop drinking alcohol since the beginning of the year because of my IBD. I love the taste of it, but I get so sick afterward. Looks like I’ll be sticking with water.
	• I saw people talking about the low-FODMAP diet. That diet was truly the most awful thing I’ve gone on.
IBD procedures	• I have Crohn’s. I got the surgery because they found that the nerves in my colon and rectum don’t function. Dealing with it all was awful.
	• Feeling emotional after this. I’m also scared for my surgery.
Marijuana	• It makes me so mad! We could’ve made medical marijuana legal for Crohn’s, but it was rejected by congress.
	• I need to see a doctor since I need medicinal weed for my IBD symptoms. My doctor isn’t the best though so I’m going to finally try and find a new one who understands how much all of this sucks.
Stigma	• I’m trying to move past the stigma of discussing digestive and reproductive issues with doctors. I have UC and I just feel uncomfortable about it.
	• Can’t we make bowel problems less awkward because everybody poops… so many people have IBD and IBS. It shouldn’t be taboo to discuss what’s normal.
Ostomy	• The incredible fear of needing another ostomy placement. IBD is so awful.
	• I’ll soon get my ostomy reversed. I’m currently excited, skeptical, and nervous…
Intimacy	• I have Crohn’s and got over my bathroom fears. I get out a lot more, but I don’t want to date anyone because I can’t stand having to discuss my IBD with them.
	• Ugh, what’s worse than your stomach making noises on a date? Has anyone else with IBD experienced similar situations while dating?

Abbreviation: IBD, inflammatory bowel disease. Text from example social media posts is paraphrased to protect the privacy and confidentiality of users.

**Table 4.** Proportion of IBD topics in the Reddit (*N* = 40 625) and Twitter (*N* = 40 306) data sets

	Reddit	Twitter
	*n* (%)	*n* (%)
Full data set		
At least 1 topic present	32 189 (79%)	22 398 (56%)
No topic present^a^	8436 (21%)	17 908 (44%)
Individual IBD topic^b^		
Symptoms	23 294 (57%)	14 488 (36%)
Medication	12 218 (30%)	4515 (11%)
Nutrition	11 039 (27%)	3675 (9%)
IBD-related procedures	6798 (17%)	2220 (6%)
Marijuana	3352 (8%)	1912 (5%)
Stigma	1482 (4%)	1250 (3%)
Ostomy	1346 (3%)	1179 (3%)
Intimacy	930 (2%)	141 (<1%)
Single and multitopic posts		
1 topic	13 705 (43%)	16 258 (73%)
2 topics	11 134 (35%)	5360 (24%)
3+ topics	7350 (22%)	780 (3%)

Abbreviations: IBD, inflammatory bowel disease; *n*, sample size.

^a^Social media posts about IBD and distress that did not contain keywords matching any one of the 8 IBD dictionary topics.

^b^Individual topics are not mutually exclusive so the aggregate percent for all topics can be greater or less than 100.

### IBD Topic Co-occurrences

**Figure 1.**
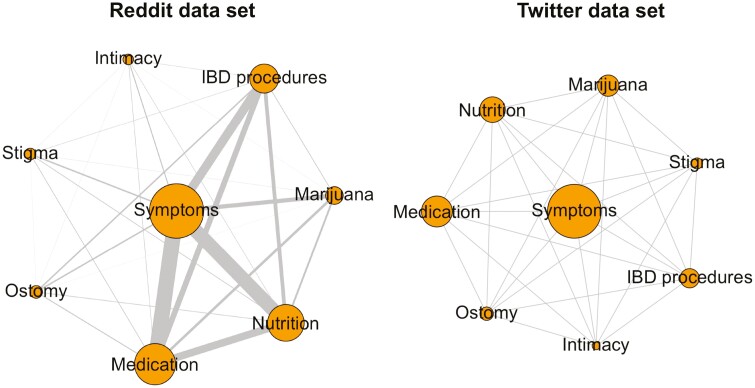
Network sociograms of topic co-occurrences for the Reddit (*N* = 18 484) and Twitter (*N* = 6140) data sets.

IBD topic co-occurrences varied by social media platform. Most Reddit posts (57%; Table 4) contained 2 or more topics. By contrast, only 27% of tweets contained multiple topics. Among multitopic social media posts, results showed that *symptoms* was the most integral topic (based on in-degree centrality) for both Reddit and Twitter data sets. For Reddit, *symptoms* co-occurred with *medication* most frequently, followed by *nutrition*. For Twitter, *symptoms* co-occurred with *medication* and *nutrition* similarly. The least integral topic among multitopic posts for both social media data sets was *intimacy*. Sociograms representing the distribution of topic co-occurrences are shown in Figure 1. Matrices containing the frequencies and proportions of topic co-occurrences, as well as in-degree centrality network statistics for each IBD topic for the Reddit and Twitter data sets can be found in Supplementary Material.

## Discussion

This study sought to identify key topics present among discussions of IBD and distress on social media. Drawing from over 80 thousand posts with results spanning 2 years, this work shows that *symptoms*, *medication*, and *nutrition* were the most prominent topics among distress-related posts about IBD on both Reddit and Twitter. In addition, this work found that the *symptoms* topic acted as a central hub among multitopic posts, highlighting its fundamental importance among IBD and distress-related social media discourse. Broadly, findings from this study add to a growing body of research investigating how IBD is discussed on social media.^8,10,14,21,31,32^ Moreover, this work expands upon the literature by empirically examining Reddit and Twitter IBD discourse in a distress-related context, thus positioning this study to be among the first to identify key areas potentially associated with IBD and experienced distress.

One of the major findings from this work was the varying degree to which discussions of IBD and distress on Reddit and Twitter mentioned different IBD topics. For example, this study showed that the IBD topics *symptoms*, *nutrition*, and *medication* are proximal to distress in online IBD discourse. In fact, 73% of Reddit posts and 48% of tweets mentioned at least one of those 3 topics. This finding may be explained due to the fact that disease activity tends to be associated with poorer distress and quality of life outcomes among people with IBD.^33–37^ Thus, it is likely that users vocalizing their IBD-related distress online are doing so as a result of symptom activity and associated irritation. IBD symptoms are also affected by disease self-management behaviors, such as adherence to medication and a symptom mitigating diet. This may explain why both *medication* and *nutrition* were also prominent topics in the Reddit and Twitter data sets, as well as why these 2 topics frequently co-occurred with *symptoms*.

A surprising finding from this research was how infrequent discussions of stigma were among the social media data sets. While evidence suggests people with IBD often report experiencing disease-related stigma,^3^ only 4% of Reddit posts and 3% of tweets discussed this topic. One explanation for this finding could be that people with IBD functionally use social media as a means to escape the experiences of offline stigma, and to instead seek/offer support and to share personal experiences among a network of other likeminded users (ie, IBD patients), as evidenced by previous work.^7,15^ Thus, it is possible that discussing stigmatizing aspects of life with IBD on social media would trigger unwanted distress or defeat the purpose of using the affordances of online communities to establish safe spaces for people with IBD.

The social media findings also showed a moderately sized discourse concerning IBD, distress, and the topic *marijuana*. Approximately 8% and 5% of posts on Reddit and Twitter mentioned at least 1 keyword from the *marijuana* dictionary. While analyzing the underlying themes of this discourse was outside the scope of the current study, it is possible that the purpose of some of these posts was to seek advice about the medicinal properties associated with marijuana (see Table 3 for example social media posts categorized under the *marijuana* topic). The therapeutic use of cannabis (ie, marijuana) to treat IBD has been discussed in the literature,^38^ and self-reported evidence from studies suggest people with IBD experience beneficial outcomes from using marijuana for symptom relief^39,40^; though, evidence is still developing in this area. Given that findings from this study indicate marijuana and IBD are being discussed on social media, future work should aim to investigate the specific nature of this discourse, such as by examining audience reception and attitudes toward cannabis as a potential therapeutic tool.

Notably, results from this work hint at potential platform effects among online IBD discourse. For instance, the topics *IBD procedures*, *ostomy*, or *intimacy* were present in only 8% of tweets, whereas the same topics appeared in nearly one-fifth (19%) of Reddit posts. This discrepancy may be due to the varying technological affordances these 2 platforms offer users. Twitter, for example, is a relatively public-facing online platform. Because of this, people with IBD may be less likely to publicly disclose or discuss stresses concerning taboo topics, such as ostomy bags or their personal relationships on Twitter. Reddit, on the other hand, is structured around the use of topic-based communities (ie, subreddits) meaning that audience members in these communities likely have shared interest or connection with one another. As such, discussing topics such as colonoscopy prep or ostomy maintenance may be commonplace and accepted unreservedly among subreddits such as r/UlcerativeColitis or r/ostomy, which supports research from other IBD and social media investigations.^7,10,15^

It should also be stated that posts on Twitter are limited by a 280-character count and it is possible that this restriction influenced the proportion of IBD topics identified by our dictionary analysis in the Twitter data set. Reddit is far less restrictive in submission and comment length, which likely explains why 57% of Reddit posts discussed multiple IBD topics compared to only 27% of tweets meeting the same criterion. This discrepancy brings to light a number of important research questions. For example, how do different platform features, such as anonymity and publishing medium (eg, text vs image vs video), affect IBD discourse on social media? More research examining the effects of affordances on this type of discourse in a multiplatform comparison is warranted and would add further insight into the purposive roles that different social media sites may play in managing IBD.

One last contribution that this study makes is that the data under investigation were from Reddit and Twitter, which are both under-researched social media platforms in the IBD literature. To date, most published IBD social media content analysis work has examined discourse on platforms such as YouTube^31,32^ and Facebook,^13,14^ and largely in the context of what type of support (ie, informational, emotional) these platforms facilitate. No studies that we are aware of have examined IBD on Reddit, positioning this research to be the first to shed light on how this disease is discussed on a popular, community-based social media platform. And while some work has examined the presence of general IBD content on Twitter, such as hashtag analysis,^8,9,21,41^ this research substantively expands on that work by systematically exploring the presence of and relationships among IBD topics in Twitter discourse. In addition, our approach to analyzing the Twitter data did not rely on hashtags alone. Instead, the text analysis methods applied in this study assessed the full text of the corpus of tweets, thus allowing a more robust and complete indication of how IBD was discussed on this particular social media platform during our data collection time period.

Strengths of this work include the use of 2 big data sets from separate social media platforms (Reddit and Twitter), a long data collection period, and robust computational procedures applied to the term-based dictionary analysis. A limitation of this study is that we were only able to analyze public Reddit and Twitter posts. Because of this, results may not reflect how IBD is discussed among users on these platforms who have private accounts. Another limitation has to do with our method for filtering for distress-related social media posts. Distress is a complex phenomenon, and it is possible that our filtering process did not capture all posts related to IBD and distress. For example, posts where distress may be implied in subtext (eg, “managing my IBD is just so fun”) may have been missed during the distress filtering step. The same limitation can be said for some of the IBD topics in the dictionary analysis, such as *stigma* and *intimacy* which are both complex and are often paired with context cues that our dictionary classification method was not equipped to identify. Future studies looking to expand on this work might wish to use other methods to filter for distress-related posts or to identify complex IBD topics, such as by using a supervised machine learning algorithm and training a computer to capture certain nuances in language.

## Conclusions

Social media platforms remain important tools for people with IBD to express themselves, seek support, and to network with others about their illness. This is particularly true for those with IBD who may feel isolated or who struggle coping with their illness. Findings from this study suggest that both Reddit and Twitter can serve as an outlet for people with IBD to express their disease-related distress across a number of important topics. Future work should expand our findings by testing the moderating role that IBD-specific online communities (as well as individual opinion leaders, such as prominent IBD advocates) play in offering support and resources to people with IBD compared to the support offered in public online discourse.

## Supplementary Material

otab044_suppl_Supplementary_Materials

## Data Availability

The data sets reported on in this manuscript are available from the corresponding author upon reason and request.
