# Supplementary material for: Topics Analysis of Reddit and Twitter Posts Discussing Inflammatory Bowel Disease and Distress From 2017 to 2019
Source: Crohns Colitis 360. 2021 Jul 7;3(3):otab044. doi: 10.1093/crocol/otab044 (PMC9802272; doi:10.1093/crocol/otab044)
Supplement: otab044_suppl_Supplementary_Materials [file otab044_suppl_supplementary_materials.docx]

# Supplemental material

**Dictionary topics and keywords**

Topic 1: Symptoms

- Symptoms_exact = ['ache', 'acid reflux', 'am week ', 'bleeding', 'bloat', 'bloody', 'changing weight', 'complications', 'constipat', 'cramp', 'diarea', 'diareah', 'diarhea', 'diarrhea', 'diarrhoea', 'digest issue', 'digest problem', 'digesting issue', 'digesting problem', 'digestive issue', 'digestive problem', 'discomfort', 'diverticulit', 'dont have energy', 'dont have the energy', 'dysphagia', 'energy disappear', 'energy is depleat', 'energy is gone', 'energy is gone', 'energy is nonexistent', 'energy is sapped', 'exhaust', 'fart ', 'farting ', 'farts ', 'fatigue', 'feel weak ', 'fever', 'fissur', 'flair up', 'flare', 'flatulen', 'fluctuating weight', 'gain weight', 'gain weight', 'gained some weight', 'gained weight', 'gaining some weight', 'gaining weight', 'gaining weight', 'gerd', 'granuloma', 'headach', 'hurt', 'im week ', 'inflamed', 'inflammation', 'insomnia', 'issue with digest', 'issue with sleep', 'issues with digest', 'issues with sleep', 'lack of energy', 'lose weight', 'losing appetite', 'losing some weight', 'losing weight', 'losing weight', 'loss of appetite', 'lost appetite', 'lost some weight', 'lost weight', 'lost weight', 'low energy', 'mucus', 'muscle stiff', 'nause', 'no appetite', 'no energy', 'pain', 'physical problem', 'problem with digest', 'problem with sleep', 'problems with sleep', 'sleep issue', 'sleep problem', 'sore', 'stomach problem', 'symptom', 'tired', 'tiring', 'ulcer ', 'ulcers ', 'upset stomach', 'upset tummy', 'vomit', 'weight change', 'weight fluctua', 'weight gain', 'weight gain', 'weight loss', 'weight loss']
- Symptoms_nonMatch = ['feel your pain', 'tired of', 'ulcers colit', 'ulcer colit', 'pain med', 'pain free', 'pain in the ass', 'painful', 'pain in the arse', 'pain in the butt', 'feveris', 'feverroot', 'feverw', 'pain kill', 'painless', 'painstak', 'paint', 'a symptomatic', 'asymptomatic', 'retired', 'pain dr', 'pain doctor', 'good pain', 'pain clinic', 'adrenal fatigue', 'not painful', 'pain relief', 'pain reliever', 'kind of a pain']

Topic 2: Medication

- Medication_exact = [' med ', ' meds ', ' pill ', ' pills ', 'acetaminophen', 'adalimumab', 'advil', 'aleve', 'aminosalicylate', 'anti inflammatories', 'anti inflammatory', 'antibiotic', 'antiinflammatories', 'antiinflammatory', 'asacol', 'asacol', 'azasan', 'azathioprine', 'balsalazide', 'biologic', 'budesonite', 'citrucel', 'codeine', 'colazal', 'corticosteroid', 'cyclosporine', 'delzicol', 'diclofenac', 'dipentum', 'entyvio', 'gengraf', 'golimumab', 'humira', 'ibuprofen', 'immodium', 'imuran', 'infliximab', 'infusion', 'injections', 'lactaid', 'lialda', 'medicat', 'medicine', 'mercaptopurine', 'mesalamine', 'methotrexate', 'methylcellulose', 'metronidazole', 'midol', 'miralax', 'motrin', 'naproxen', 'narcotic', 'natalizumab', 'neoral', 'nsaid', 'olsalazine', 'paracetamol', 'pentasa', 'prednisone', 'purinethol', 'purixan', 'remicade', 'sandimmune', 'simponi', 'stelara', 'stelera', 'steroid', 'suppositor', 'thioguanine', 'trexall', 'tylenol', 'tysabri', 'uceris', 'ustekinumab', 'vedolizumab', 'voltaren']
- Medication_nonMatch = ['aleveate', 'spill', 'medicine unit', 'anti inflammatory diet', 'antiinflammatory diet', 'hair injections']

Topic 3: Nutrition

- Nutrition_exact = [' bran ', ' egg', ' fish ', ' gin ', ' lime', ' meal ', ' meal ', ' meals ', ' meals ', ' meat', ' oats', ' pear', ' peas ', ' rice', ' rum ', ' rye ', ' soy ', 'alcohol', 'almond', 'apple', 'apricot', 'artichoke', 'arugula', 'asparagus', 'avocado', 'banana', 'barley', 'beer', 'bok choy', 'bread', 'broccoli', 'butter', 'cabbage', 'caffei', 'calcium', 'calori', 'candies', 'candy', 'cantaloupe', 'carbohydrate', 'carbs', 'carrot', 'cashew', 'cauliflower', 'cheese', 'chicken', 'cognac', 'collard', 'corn ', 'cucumber', 'dairy', 'diet', 'edamame', 'eggplant', 'electrolyt', 'fasting', 'fatty acid', 'fiber', 'fodmap', 'folate', 'food', 'fruit', 'gluten free', 'glutenfree', 'grape', 'greasy', 'green bean', 'hazelnut', 'herring', 'high fat', 'highfat', 'hydrat', 'juice', 'kale', 'kefir', 'kimchi', 'kiwi', 'lemon', 'lettuce', 'liqor', 'liquor', 'low fat', 'low fiber', 'lowfat', 'lowfiber', 'lowfodmap', 'lychee', 'mackerel', 'magnesium', 'mannitol', 'margarin', 'melon', 'milk', 'millet', 'mineral', 'miso', 'noodle', 'oatmeal', 'okra', 'onion', 'paleo', 'paleolithic', 'papaya', 'pasta', 'peanut', 'pecan', 'pepper', 'pineapple', 'pistachio', 'plum', 'polyol', 'popcorn', 'pork', 'potassium', 'potato', 'poultry', 'protein', 'quinoa', 'saccharide', 'salmon', 'sardine', 'sauerkraut', 'sesame seed', 'soda', 'sorbitol', 'sourdough', 'spelt', 'spice', 'spicy', 'spinach', 'sprouts', 'sugar', 'sunflower seed', 'tempeh', 'tequila', 'tequilla', 'tofu', 'turkey', 'vegetable', 'vermouth', 'vodka', 'walnut', 'watercress', 'wheat', 'whiskey', 'whisky', 'wine', 'yoghurt', 'yogurt', 'zucchini']
- Nutrition_nonMatch = ['spice girl', 'pizza express', 'blood sugar', 'nutritionist', 'chickened', 'apple iphone', 'apple product', 'greapevine', 'spicegirl', 'fiberglass', 'fiber glass', 'fiberboard', 'fiber optic', 'fiberoptic', 'fiberotom', 'fiberscop', 'fruitful', 'milkiest', 'milkmaid', 'milky', 'food service', 'go bananas', 'alcoholism']

Topic 4: IBD procedures

- IBD_procedures_exact = ['anastomosis', 'colectomy', 'colonosco', 'endoscop', 'post op ', 'post operati', 'postop ', 'procedure', 'proctocolectomy', 'resection', 'strictureplasty', 'surgeri', 'surgery', 'surgical', 'tracheostom', 'transplant']

Topic 5: Marijuana

- Marijuana_exact = [' thc ', 'bong', 'canna butter', 'cannab', 'cbd', 'dab', 'dabbed', 'edible', 'hash', 'herb', 'indica ', 'marijuana', 'mary jane', 'maryjane', 'sativa', 'stoner', 'strawberry cough', 'weed']
- Marijuana_nonMatch = ['rehash', 're hash']

Topic 6: Stigma

- Stigma_exact = [' bias ', ' biased', 'ableism', 'ableist', 'bully', 'demean', 'discriminat', 'disrespect', 'embarrass', 'faked it', 'faking it', 'humiliat', 'its no joke', 'its not a joke', 'labeling', 'made fun of', 'make fun of', 'making it up ', 'misconstru', 'misinterpret', 'misknown', 'misperceiv', 'mispercept', 'mock ', 'mocked', 'mocking', 'poke fun', 'poked fun', 'prejudice', 'ridicule', 'single out', 'singled out', 'stereoty', 'stigma', 'teased', 'treat different', 'treat unfair', 'treated different', 'treated unfair', 'type cast', 'typecast', 'victimiz']

Topic 7: Ostomy

- Ostomy_exact = ['colostomy', 'ileostomy', 'illeostomy', 'jpouch', 'j pouch', 'ostomate', 'ostomy', 'stoma ', 'wafer ']

Topic 8: Intimacy

- Intimacy_exact = [' dating ', ' hinge ', ' sex ', 'blind date', 'boink', 'bumble', 'canoodl', 'coffee meets bagel', 'coffeemeetsbagel', 'coitus', 'copulat', 'doing the dirty', 'fornic', 'frisky', 'grindr', 'hook up', 'hooking up', 'intamacy', 'intamate', 'intercourse', 'intimacy', 'intimate', 'kiss', 'knocking boot', 'made out with', 'make out with', 'making love', 'making out with', 'making whoop', 'mating', 'ok cupid', 'okcupid', 'on a date', 'penetration', 'plenty of fish', 'plentyoffish', 'ravish', 'romance', 'romancing', 'romantic', 'scissoring', 'scissr', 'seduce', 'sexual relation', 'shag', 'smooch', 'spooning', 'tinder', 'valentine'

**Network statistics for co-occurring IBD topics**

Results in Table S1 shows the undirected degree centrality values for the IBD topics networks for both Reddit and Twitter. Degree centrality refers to the number of connections that a node (i.e., an IBD topic) has to other nodes in a network and is used as an indicator of node interconnectedness. Higher degree centrality values for an IBD topic indicate that said topic was more central to social media posts discussing multiple IBD topics. Notably, topics networks are comprised only of posts that contain two or more IBD topics. Results in Tables S2 and S3 show the unadjusted frequency and proportion of IBD topic co-occurrences in social media posts.

**Table S1.** Degree centrality values among IBD topics on Reddit and Twitter.

| **IBD Topic** | Reddit | Twitter |
| --- | --- | --- |
| Symptoms | 25,103 | 5,719 |
| Medication | 17,978 | 2,953 |
| Nutrition | 15,602 | 2,306 |
| IBD procedures | 11,448 | 1,536 |
| Marijuana | 5,371 | 1,793 |
| Stigma | 2,080 | 540 |
| Ostomy | 2,693 | 833 |
| Intimacy | 1,593 | 96 |

*Note.* Cell values in columns represent each topic’s degree centrality value (i.e., the number of connections the IBD topic had with other topics).

**Table S2.** Frequency of IBD topic co-occurences among the Reddit data set; *N*=40,625

| **Topic** | Symptoms  *n* (%) | Medication  *n* (%) | Nutrition  *n* (%) | Procedures  *n* (%) | Marijuana  *n* (%) | Stigma  *n* (%) | Ostomy  *n* (%) |
| --- | --- | --- | --- | --- | --- | --- | --- |
| Medication | 8,332 (21%) | — |  |  |  |  |  |
| Nutrition | 7,511 (18%) | 4,051 (10%) | — |  |  |  |  |
| IBD procedures | 4,775 (12%) | 3,044 (7%) | 2,012 (5%) | — |  |  |  |
| Marijuana | 2,287 (6%) | 1,392 (3%) | 1,030 (3%) | 385 (1%) | — |  |  |
| Stigma | 810 (2%) | 363 (1%) | 386 (1%) | 230 (1%) | 127 (<1%) | — |  |
| Ostomy | 806 (2%) | 519 (1%) | 356 (1%) | 785 (2%) | 71 (2%) | 69 (<1%) | — |
| Intimacy | 582 (1%) | 277 (1%) | 256 (1%) | 217 (1%) | 79 (2%) | 95 (<1%) | 87 (<1%) |

*Note. n*=sample size.

**Table S3.** Frequency of IBD topic co-occurences among the Twitter data set; *N*=40,306

| **Topic** | Symptoms  *n* (%) | Medication  *n* (%) | Nutrition  *n* (%) | Procedures  *n* (%) | Marijuana  *n* (%) | Stigma  *n* (%) | Ostomy  *n* (%) |
| --- | --- | --- | --- | --- | --- | --- | --- |
| Medication | 1,772 (4%) | — |  |  |  |  |  |
| Nutrition | 1,423 (4%) | 380 (1%) | — |  |  |  |  |
| IBD procedures | 783 (2%) | 326 (1%) | 120 (<1%) | — |  |  |  |
| Marijuana | 1,120 (3%) | 329 (1%) | 246 (1%) | 40 (<1%) | — |  |  |
| Stigma | 236 (1%) | 71 (<1%) | 43 (<1%) | 57 (<1%) | 39 (<1%) | — |  |
| Ostomy | 347 (1%) | 67 (<1%) | 87 (<1%) | 205 (1%) | 16 (<1%) | 85 (<1%) | — |
| Intimacy | 38 (<1%) | 8 (<1%) | 7 (<1%) | 5 (<1%) | 3 (<1%) | 9 (<1%) | 26 (<1%) |

*Note. n*=sample size.
